# Fabrication and Characterization of Zein/Sodium Alginate Nanoparticles Loaded with Oxyresveratrol: Formation Mechanism, Molecular Dynamics Insights, and In Vitro Antioxidant Capacity

**DOI:** 10.3390/mi17050594

**Published:** 2026-05-13

**Authors:** Xiaomin Luo, Zhiyun Du

**Affiliations:** School of Biomedical and Pharmaceutical Sciences, Guangdong University of Technology, Guangzhou 510006, China

**Keywords:** nanotechnology, Oxyresveratrol, zein, self-assembly, molecular dynamics

## Abstract

Oxyresveratrol (Oxy) exhibits a diverse range of biological activities. However, its practical application is constrained by low aqueous solubility and chemical instability. In this work, Oxy-loaded zein (Z) nanoparticles (NPs) stabilized by a sodium alginate (Alg) coating (Oxy-Z/Alg NPs) were fabricated using an antisolvent precipitation method. The absence of crystalline peaks in X-ray diffraction analysis suggested that Oxy was dispersed as an amorphous phase in NPs, while the Fourier transform infrared spectra identified strong interfacial associations between the components. The stabilization of the NPs is attributed to the site-specific binding of Oxy with Z’s SER-162 and GLN-174 residues. Molecular docking, molecular dynamics simulations, and differential scanning calorimetry profiles evidenced the formation of intermolecular hydrogen bonds. Dynamic light scattering analysis showed that the nanocomplexes had a nano-scale dimension (243 ± 6 nm) and a zeta potential of −36 mV. SEM micrographs revealed that the NPs possessed a spherical morphology. The NPs exhibited colloidal stability against prolonged heating (80 °C for 75 min), ionic strengths (up to 100 mM NaCl), and pH range (2.0–10.0). Encapsulation within the Alg coating enhanced Oxy’s antioxidant capacity over its unprotected form by shielding its core bioactivity from degradation. The Oxy-Z/Alg nano-system shows significant promise for the encapsulation of Oxy, providing a practical basis for its integration into nutraceuticals and functional food fields.

## 1. Introduction

Oxyresveratrol (Oxy) is a prominent stilbenoid phytochemical found in Vitis vinifera pericarp and Fabaceae plants for its diverse biological activities [1]. It possesses a vast variety of pharmacological effects, including neuroprotective, radical-scavenging, anti-inflammatory, anti-diabetic, and anti-tumor potencies [2] and has gained academic interest. Nevertheless, Oxy is naturally lipophilic and its molecular nature is not stable. It can also be easily affected by various harsh conditions inside the gastrointestinal tract, making it hard to be widely employed in the food and drug industries [3]. To address these limitations, nanoscale encapsulation has emerged as a prominent strategy. This technology has garnered widespread attention due to its small particle size and high encapsulation capacity for active molecules [4]. Recently, researchers have conducted a great number of investigations on biocompatible protein-based materials. Among these, nanoparticles (NPs) constructed from zein (Z) have drawn particularly wide attention. Z NPs are often chosen as suitable carriers to encapsulate different kinds of bioactive compounds, mainly because they have been proven safe for biological systems and can show reliable performance during substance delivery [5]. Being a primary prolamin stored in corn, Z protein can be divided into four different fractions, namely ɑ-, β-, γ-, and δ-. Across these types, α-Z is the most abundant and also commonly employed in relevant work [6]. Owing to its particular structure, α-Z possesses special amphiphilic features. Nonpolar amino acid residues make up a large part of its molecular structure, with leucine, alanine, and proline being the main ones. It also contains relatively few polar residues, a structural attribute that inherently leads to clearly amphiphilic behavior within the molecule [7]. This nonuniform arrangement of polarity facilitates self-assembly of the Z protein on a molecular scale [8], allowing it to associate strongly with polyphenolic compounds [9]. For example, after encapsulation, the resulting Z composites preserve curcumin and allow its functional activities to be realized [10]. However, Z NPs have a strong tendency to aggregate into large clusters when exposed to environments with their isoelectric pH. Their intrinsic instability in aqueous environments also poses a barrier to their integration into commercial food and pharmaceutical formulations [11]. To address these problems, one common strategy is to coat the surface of Z NPs with some biopolymers that help keep them stable.

Sodium alginate (Alg) is derived from marine organisms. It possesses polyanionic properties and exhibits excellent water-binding capacity [12]. Its surface carries a strong negative charge, and this structural feature allows electrostatic interactions to step in [13]. By utilizing these attributes, interfacial coating of Z cores with Alg constructs a strong barrier, effectively enhancing the colloidal stability [14]. Encapsulating phytosterols within the coated Z NPs effectively improves the bioactives’ aqueous solubility and structural stability [15]. Likewise, the incorporation of cinnamaldehyde and α-mangostin into polysaccharide-functionalized Z-based nanocomplexes yields pronounced enhancement in both antibacterial efficacy and antioxidant activity [16]. Compared with binary Z systems, these ternary nanostructures exhibit superior stability and radical scavenging performance [17]. Numerous studies have examined the application and characteristics of nanocarriers made from Z. But the potential of integrating Alg to enhance the encapsulation performance of Oxy molecules has not been studied.

As far as we know, this study is the first to report the ternary interaction mechanism among Oxy, Alg, and Z. After optimizing the formulation parameters systematically, we comprehensively evaluated the physicochemical properties of Oxy-loaded Z/Alg NPs, including encapsulation rate, loading capacity, and microscopic morphology. To understand the molecular mechanisms of the assembly among Z, Oxy, and Alg, their intermolecular interactions and crystalline states were assessed, including Fourier Transform Infrared (FTIR) spectroscopy, X-ray diffraction (XRD), and differential scanning calorimetry (DSC). Subsequently, molecular docking and molecular dynamics simulations were utilized to reveal the binding modes and structural stability of the Oxy-loaded Z/Alg system. We then assessed the environmental stability and antioxidant capacity of the delivery system under various external conditions, including thermal stress, pH, and saline environments. The current work provides a fundamental theoretical basis for the construction of stable delivery platforms of Oxy.

## 2. Materials and Methods

### 2.1. Materials

Oxyresveratrol (Oxy) was given by Sun Yat-sen University in Guangzhou, China. Zein (Z), and 2,2-diphenyl-1-picrylhydrazyl (DPPH), and 2,2′-azino-bis(3-ethylbenzothiazoline-6-sulfonic acid) (ABTS) were sourced from Macklin Biochemical Co., Ltd. (Shanghai, China). Sodium alginate (Alg, analytical grade, Cat# S817374) was obtained from Macklin (Shanghai, China). The manufacturer reported a viscosity range of 200–550 cp (measured as a 1% aqueous solution at 20 °C), which corresponds to an estimated average molecular weight of approximately 220–350 kDa based on the empirical relationship between viscosity and chain length for this polymer. All supplementary reagents and chemicals utilized were all of analytical grade in this study.

### 2.2. Fabrication of NPs

The Oxy-loaded Z/Alg NPs were prepared using an antisolvent precipitation method, adapted from earlier literature with minor modifications [18]. In brief, Oxy and Z (0.2 g) were combined at a mass ratio set to 1:3.2 and then co-dissolved in 8 mL of 80% (v/v) ethanol–water mixture. The mixture was mechanically shaken at 850 rpm for 60 min. To fabricate the Oxy-Z NPs, the above solution was added to 32 mL of purified water under continuous stirring, then rotary evaporated at 40 °C to remove ethanol.

The preparation of Oxy–Z/Alg NPs mirrored the above procedure, except for replacing purified water with Alg aqueous solutions. Five different Z-to-Alg weight ratios were adopted: 20:1, 10:1, 5:1, 2.5:1, and 1.25:1.

### 2.3. Measurement of Z-Average Hydrodynamic Diameter, Polydispersity, and Zeta Potential

The Z-average hydrodynamic diameter, polydispersity index (PDI), and zeta potential of NPs were examined using a Brookhaven BI-200SM dynamic light scattering system (DLS, New York, NY, USA) at 25 °C. The particle size and PDI were calculated via Cumulants analysis. All experimental data were collected from three separately prepared batches (*n* = 3). All measurements were performed on three independently prepared batches, and the data are presented as mean values and their standard deviations.

### 2.4. Measurement of Encapsulation Performance

The measured encapsulation efficiency (EE) and loading capacity (LC) of the NPs were derived from a previously validated methodology [19]. Initially, samples were equilibrated in acetonitrile under sonication for 10 min, followed by centrifugation at 12,000 rpm to segregate the insoluble debris and collect the purified supernatant. Following a 15 min interval, the resulting supernatant was quantified via an HPLC platform (Thermo Fisher Scientific, Waltham, MA, USA) equipped with a reversed-phase ODS-C18 column. Target analytes were monitored at 320 nm. A constant oven temperature of 35 °C was applied to the column, while a binary solvent matrix consisting of acetonitrile and deionized water (60:40, v/v) was employed. Quantitation of Oxy was executed through the Xcalibur software (V.3.0) interface (Thermo Scientific). The subsequent equations were applied to measure the EE and LC:

(1) $$\mathrm{EE}\;(\%)\;=\;\frac{Mass\;of\;encapsulated\;Oxy}{Total\;mass\;of\;Oxy\;added}\;\times \;100$$

(2) $$\mathrm{LC}\;(\%)=\frac{Mass\;of\;encapsulated\;Oxy}{Total\;mass\;of\;NPs}\;\times \;100$$

### 2.5. Characterization

#### 2.5.1. Morphological Features

The morphology of the NPs was observed using a field emission scanning electron microscope (FE-SEM, Zeiss Sigma 500, Oberkochen, Germany). Specimens were given a thin gold coating via sputtering to provide electrical conductivity. Secondary electron signals were then recorded under a 10.0 kV accelerating voltage to acquire the images.

#### 2.5.2. X-Ray Diffraction (XRD) Analysis

To elucidate the structural crystallinity, XRD profiles were recorded on a Bruker D8-Advance system (Bruker, Karlsruhe, Germany) equipped with a Cu radiation source. The angular range was recorded over an angular range of 5° to 40° (2 degrees). The scanning speed was set to 5° per minute.

#### 2.5.3. Fourier Transform Infrared (FTIR) Spectroscopy Analysis

FTIR spectra were collected via a Nicolet iS50R instrument (Thermo Fisher Scientific, USA) to explore the intermolecular interactions within the NPs. Spectra data were corrected between 4000 and 400 cm^−1^ with a 4 cm^−1^ resolution.

#### 2.5.4. Secondary Structural Determination

To determine the secondary structure composition of Z and the Oxy-Z/Alg complexes, their amide I bands (1700–1600 cm^−1^) were subjected to second-derivative processing using PeakFit v4.12. Subsequently, a Gaussian deconvolution and curve-fitting procedure was carried out. The relative area contributions of each resolved component were then calculated, enabling quantification of changes in their secondary structural makeup.

#### 2.5.5. Differential Scanning Calorimetry (DSC) Analysis

The thermal characteristics of samples were investigated by differential scanning calorimetry (DSC, Perkin Elmer, Waltham, MA, USA). The procedure was as follows: 4.0 mg of each sample was placed in a standard aluminum pan, sealed tightly with a perforated aluminum cover, and then warmed from 20 to 250 °C at a constant 10 °C·min^−1^. Dry nitrogen gas was continuously flushed through the system at 20.0 mL·min^−1^.

### 2.6. Molecular Docking

Protein templates (UniProt: Q41844) and ligand structures (TCMSP/PubChem) were retrieved for in silico docking [20]. Following PDBQT conversion, AutoDock Vina 1.2.2 was implemented to simulate binding within a predefined active-site volume. Among nine generated trajectories, the highest-affinity poses were prioritized. Interfacial interactions were systematically dissected using PLIP (v.2.2.2)and LigPlot+ (v.2.3.1), with atomic-level visualizations rendered via PyMOL (v2.5) [21].

### 2.7. Molecular Dynamics Simulation

The dynamic behavior and structural consistency of the prepared complexes were assessed via molecular dynamics simulations run on GROMACS 2022.3. Initial steps involved using Gaussian 16 W to hydrogenate the molecular structure and derive its restrained electrostatic potential charge distribution. Assignment of the general amber force field parameters was next carried out through the AmberTools 22 suite. The obtained electronic and structural values were embedded within the exhaustive topology files meant for the final simulated system. A cubic periodic box full of TIP3P water molecules hosted the constructed complexes to create the simulation environment. Na^+^ ions were introduced into the solvent box to cancel out its residual charge, following the system’s net demand. A steepest descent approach was first applied for structural minimization to reduce atomic tension. The system underwent equilibration using both constant-volume (NVT) and constant-pressure (NPT) ensembles. Temperature regulation at 300 K was achieved via the V-rescale thermostat, set with a coupling constant $\tau_{t}$ of 0.1 ps. Simultaneously, the Parrinello–Rahman barostat kept pressure fixed at 1.0 bar using a $\tau_{p}$ value of 2.0 ps.

A steepest descent routine served to reduce the energy of the initial geometries. Two consecutive equilibration phases were then performed: first a 100 ps simulation with NVT, followed by another 100 ps with NPT. Once thermodynamic equilibrium was achieved, a 100 ns molecular dynamics production simulation proceeded without retaining any constraints on atomic positions. This production run involved 50 million iterations at a step size of 2 fs. The recorded trajectories were studied to understand the binding behavior and the flexible nature of the nanocomposite materials. System stability and structural compactness were tracked through calculations of root mean square deviation (RMSD) and radius of gyration (Rg). Meanwhile, residue-level mobility was assessed using root mean square fluctuation (RMSF) values. Total binding affinity was evaluated by applying the MM/GBSA (molecular mechanics/generalized born surface area) method for free energy estimation. Free energy landscapes (FELs) were derived to pinpoint the set of most stable conformations.

### 2.8. Evaluation of the Stability

#### 2.8.1. Evaluation of Thermal Stability

Fresh dispersions were subjected to isothermal treatment (80 °C) for 15–75 min to evaluate thermal robustness [22]. After cooling to 25 °C, particle size and zeta potential were monitored.

#### 2.8.2. Evaluation of pH Stability

To evaluate the pH-responsive behavior, the colloidal systems were equilibrated over a pH range of 2.0–10.0 using HCl and NaOH solutions [23]. After 24 h, the mean size, PDI, and zeta potential were evaluated.

#### 2.8.3. Evaluation of Ionic Strength Stability

Freshly prepared NPs were mixed with a salinity gradient (0–70 mM NaCl) via 1:1 volumetric mixing with the aqueous phase [24]. Following a 24 h incubation, the average particle size, PDI, and zeta potential were measured.

### 2.9. Evaluation of the In Vitro Antioxidant Capacity of NPs

The DPPH and ABTS radical scavenging capacities of free Oxy and Oxy-Z/Alg NPs were determined as described previously [25,26]. Samples (100 μL) were mixed with a 0.2 mM DPPH radical reagent in equal volumes. The resultant absorbance at 517 nm was measured after a 30 min dark incubation.

ATBS radical cation was generated by reacting 7 mM ABTS with potassium persulfate. The radical cation mixture was diluted with ethanol until the absorbance at 734 nm reached 0.70 ± 0.02. 100 μL of ABTS reagent was mixed with 50 μL of sample. The reaction was allowed to proceed for 10 min for complete reaction, and the absorbance was recorded at 734 nm. The radical scavenging percentage was calculated using the formula in Equation (3):

(3) $$\mathrm{Radical}\;\mathrm{scavenging}\;\mathrm{rate}\;(\%)\;\;=1-\frac{A_{s}-A_{c}}{A_{b}}\times 100$$

$A_{s}$, $A_{b}$, and $A_{c}$ correspond to the absorbance signals measured from the sample-radical mixture, blank radical solution, and sample solvent control group, respectively.

### 2.10. Statistical Analysis

Experimental assays were performed in triplicate, with results presented as mean ± SD. One-way ANOVA was performed using IBM SPSS 26.0 to compare group means. Subsequent multiple comparisons were carried out via Tukey’s HSD test, with significance set at *p* < 0.05.

## 3. Results and Discussion

### 3.1. Effect of Alg Concentration

#### 3.1.1. Particle Size, PDI, and Zeta Potential

**Figure 1:**
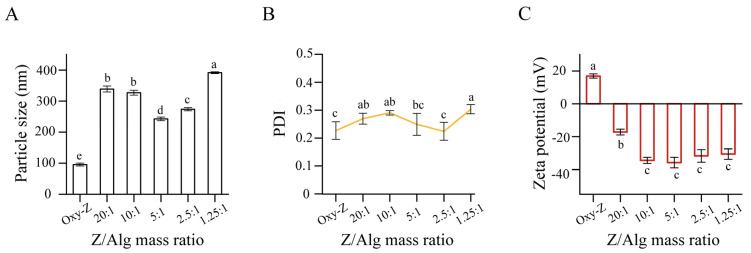
Effects of varying Z/Alg mass ratios on (**A**) mean particle size, (**B**) PDI, and (**C**) zeta potential of the complexes at a fixed Z concentration of 5.0 mg·mL^−1^ and a constant Oxy/Z mass ratio of 1:3.2. The abscissa denotes a series of Z/Alg mass ratios ranging from 20:1 to 1.25:1. Results are presented as mean ± standard deviation (*n* = 3). Distinct letter labels on the bars indicate significant group differences at the *p* < 0.05 level, as calculated using one-way ANOVA combined with Tukey’s post hoc test.

Particle diameter, PDI, and zeta potential reflect the colloidal stability and assembly efficiency of delivery systems. As shown in Figure 1A, the initial Oxy-Z binary particles had a particle size of approximately 100 nm. Upon the incorporation of Alg, the particle size significantly increased (*p* < 0.05), ranging from 240 nm to 392 nm. This confirms the development of a more complex ternary architecture [27]. At high Z/Alg ratios (20:1 and 10:1), the particle sizes exceed 325 nm, likely due to inter-particle bridging. Under these conditions, the insufficient concentration of Alg chains may partially cover multiple Oxy-Z cores, resulting in loosely bound aggregates [28]. The minimum particle size was observed at the 5:1 ratio (243 ± 6 nm). At this point, the Alg coating appears to neutralize the zeta potential and provide structural compactness via electro-steric repulsion. This likely represents the saturation point for Alg coating on the Oxy-Z cores. The PDI profiles in Figure 1B reflect the structural evolution from binary to ternary systems. The Oxy-Z NPs exhibited the narrowest size distribution. At low Alg concentration (Z/Alg ratios of 20:1 to 10:1), the PDI values slightly increased, likely due to the initial bridging. Nevertheless, when the Z/Alg mass ratios reached 5:1 and 2.5:1, the PDI progressively decreased. The subsequent decrease in PDI indicated that a more uniform polysaccharide shell formed around the particles. An increase in particle size (~392 nm) and PDI (~0.30) occurred at a 1.25:1 ratio. This likely results from depletion flocculation or secondary aggregates caused by excess Alg [29]. As illustrated in Figure 1C, the Oxy-Z binary complexes exhibited a positive zeta potential due to the protonated amino groups of Z [30]. Adding Alg reversed the zeta potential from positive to negative in all ternary formulations, suggesting that the anionic polysaccharide deposited onto the cationic protein core [31]. Specifically, when the Z/Alg ratio was decreased from 20:1 to 5:1, the absolute value of zeta potential rose from −17 mV to around −36 mV. At this 5:1 ratio, the zeta potential was high enough to meet the stability requirement, helping maintain the electro-steric stabilization of the Oxy-Z/Alg NPs. Further increasing the Alg ratio to 2.5:1 or 1.25:1 gave similar zeta potentials; this suggests that the Oxy-Z core surface reached saturation with Alg at a 5:1 ratio. Thus, the small change in zeta potential at higher Alg ratios may not substantially improve electrostatic stabilization. The 5:1 ratio appeared to achieve the highest stabilization among the tested ratios.

#### 3.1.2. Encapsulation Performance

**Figure 2:**
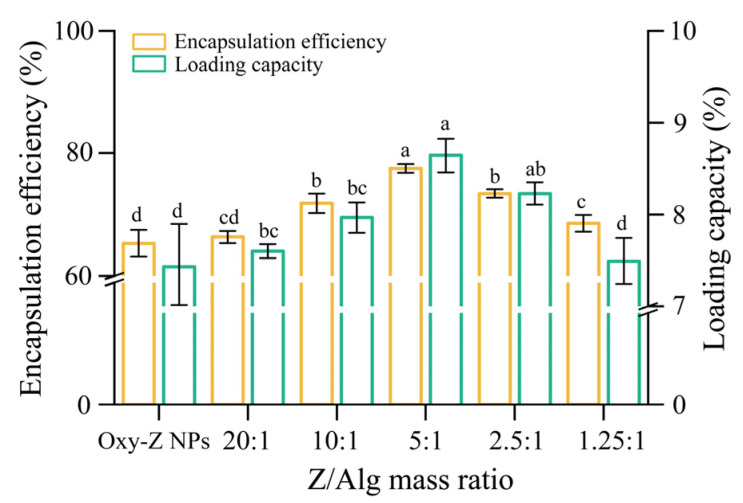
Effects of varying Z/Alg mass ratios on the encapsulation efficiency (EE) and loading capacity (LC) of Oxy within the colloidal system, with a fixed Z concentration of 5.0 mg·mL^−1^ and a constant Oxy/Z mass ratio of 1:3.2. A series of Z/Alg mass ratios (20:1 to 1.25:1) are plotted on the abscissa. EE is defined as the fraction (in percent) of initially added Oxy that becomes encapsulated. LC gives the amount of Oxy trapped inside per unit weight of collected NPs. Data are shown as mean ± standard deviation (*n* = 3). Bars marked with different letters reflect statistically significant differences among groups (*p* < 0.05), as determined by one-way ANOVA followed by Tukey’s post hoc test.

EE and LC are key parameters for evaluating colloidal delivery systems [32]. Figure 2 shows the EE and LC of the NPs. The binary Oxy-Z system without Alg had lower EE and LC than the ternary systems containing Alg. A higher Z/Alg ratio increased EE and LC from nearly 66% and 7.5% to 78.35% and 8.67%, respectively. This improvement was due to the capacity of Alg to bind pure Oxy on Z NPs surface. With a Z/Alg mass ratio of 5:1, the measured EE and LC of Oxy-Z/Alg NPs were 1.16 and 1.25 times higher than those of Oxy-Z NPs, respectively. The Alg coating decreased Z’s hydrophobicity, thereby leading to enhanced NP stability and preventing coagulation. At low Alg concentrations, the interaction between Alg and Z was too weak to form an adequate surface layer. This allowed some Oxy compounds to leak from the NPs. In contrast, NPs produced with higher Alg concentrations had better encapsulation performance. With these Alg concentrations, interactions between Alg and Z became stronger, driven by hydrophobic forces and hydrogen bonding. This led to denser NPs that encapsulated Oxy more efficiently, as shown by the higher EE and LC values. However, EE and LC decreased significantly when the Z/Alg ratio exceeded 5:1, possibly due to larger particle size at higher concentrations. Smaller nanocomplexes had a more uniform coating layer, which better protected the bioactive compositions. The NPs with the smallest size (242.8 ± 6.0 nm) also had high EE and LC. Thus, a Z/Alg mass ratio of 5:1 was selected for subsequent experiments.

### 3.2. Characterization

#### 3.2.1. FE-SEM Analysis

**Figure 3:**
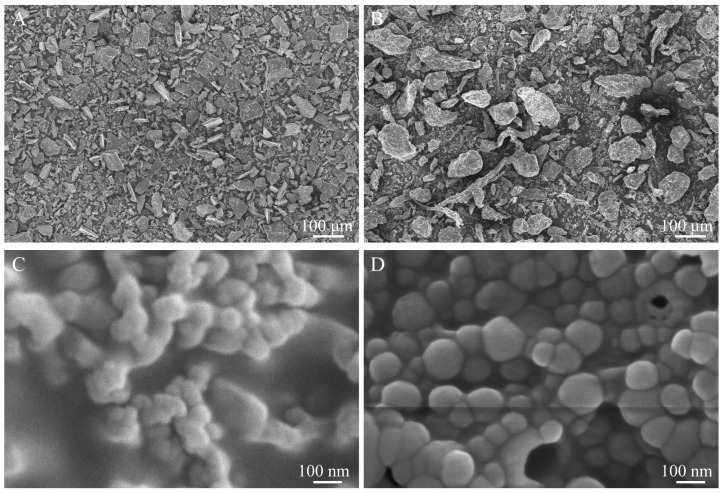
FE-SEM images of (**A**) pure Oxy, (**B**) Alg, (**C**) Z, (**D**) Oxy-Z/Alg NPs (prepared with a Z concentration of 5.0 mg·mL^−1^, an Oxy/Z mass ratio of 1:3.2, and a Z/Alg mass ratio of 5:1).

The morphology of the resulting NPs and their components was observed via FE-SEM. Pristine Oxy exhibited large, irregular crystalline aggregates with size in the micrometer range (Figure 3A). Alg exhibited an irregular and fragmented morphology characterized by a rough and flaky surface (Figure 3B). This microscopic morphology matches the conventional look of unmodified Alg powder described in earlier studies [33]. Z appeared as smooth spherical particles with diameters of approximately 60–80 nm (Figure 3C). The Oxy-Z/Alg NPs remained a well-defined spherical morphology (Figure 3D). However, their size was slightly larger than that of pristine Z NPs. The larger size indicates that Oxy was loaded into the Z cores and that Alg coated their surface. This may result from enhanced electrostatic attraction between Alg and Z. The particle sizes observed by SEM were smaller than the hydrodynamic diameters measured by DLS (Figure 1A), which is common for biopolymer-based NPs. The reason for this mismatch is that DLS reports the hydrodynamic size, which combines the solid particle core together with the solvated layer on its outer surface. By comparison, microscopic imaging shows the physical dimensions of the dried solid interior. The natural discrepancy in their respective physical meanings accounts for the generally larger readings from DLS.

#### 3.2.2. XRD Analysis

**Figure 4:**
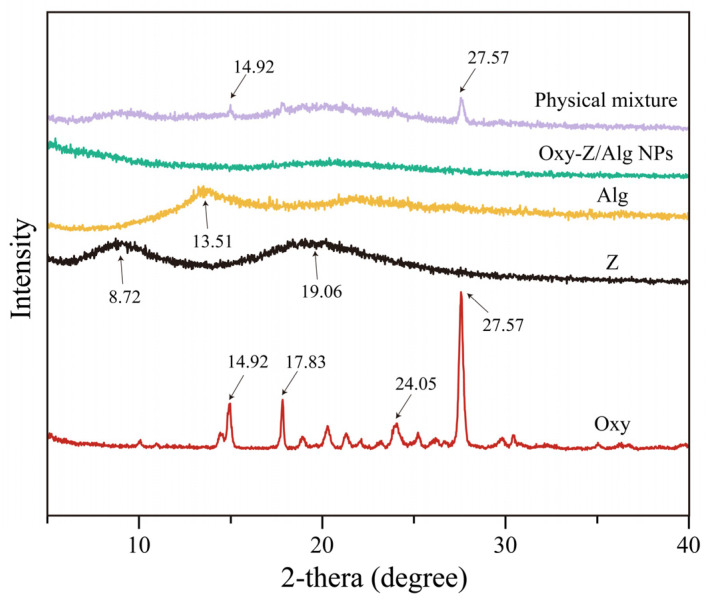
XRD spectra of Oxy, Z, Alg, physical mixture, and the Oxy-Z/Alg NPs (prepared with a Z concentration of 5.0 mg·mL^−1^, an Oxy/Z mass ratio of 1:3.2, and a Z/Alg mass ratio of 5:1).

To examine the crystalline structure of the Oxy-Z/Alg NPs and their components, XRD patterns were collected. As shown in Figure 4, pure Oxy had sharp peaks at 14.92°, 17.83°, 24.05°, and 27.57°, indicating its highly crystalline structure. Contrarily, Z showed two characteristic peaks at approximately 8.72° and 19.06°, corresponding to the inter- and intra-molecular spacing of its protein structure [34]. Similarly, Alg had a broad peak at 13.51°, typical of an amorphous polymer. The XRD pattern of the physical mixture (Z, Oxy, and Alg) was a combination of the individual components, with clear peaks from Oxy. No crystalline peaks of Oxy were observed in the ternary Oxy-Z/Alg NPs. This indicates that Oxy became amorphous likely due to the interactions with the carrier, and confirms its successful loading [35]. The lack of crystalline peaks suggests that intermolecular interactions disrupted crystallization, which may increase Oxy’s solubility and bioaccessibility.

#### 3.2.3. FTIR Analysis

**Figure 5:**
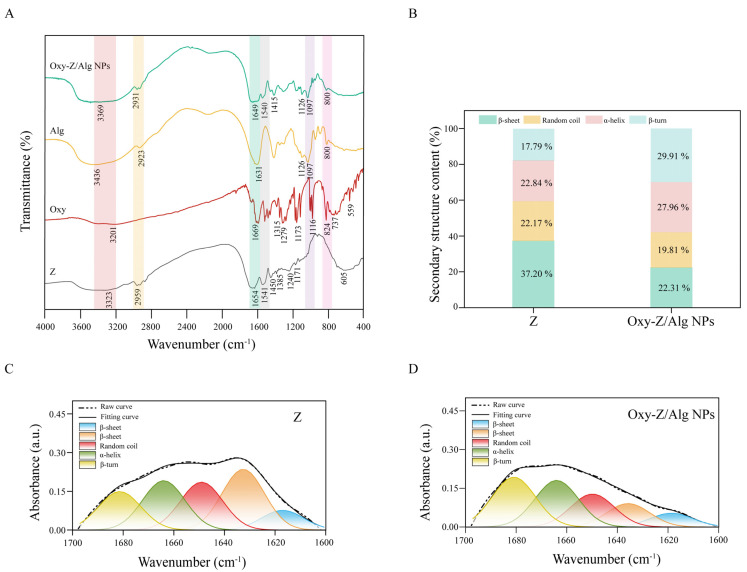
FTIR spectroscopy analysis of Z, Alg, Oxy, and Oxy-Z/Alg NPs (prepared with a Z concentration of 5.0 mg·mL^−1^, an Oxy/Z mass ratio of 1:3.2, and a Z/Alg mass ratio of 5:1). (**A**) FTIR spectra in the range of 4000–400 cm^−1^. (**B**) Comparative analysis of Z secondary structure percentages derived from the Amide I region. (**C**,**D**) Curve-fitting and deconvolution results of the Amide I band for pristine Z (**C**) and the Oxy-Z/Alg NPs (**D**), respectively.

FTIR spectroscopy was used to examine the molecular interactions in the Oxy-Z/Alg NPs. As shown in Figure 5A, the FTIR spectrum of pristine Z displays bands at 3323 cm^−1^ (assigned to N–H stretch), 1654 cm^−1^ (Amide I, C=O stretch), 1541 cm^−1^ (Amide II, N–H bending/C–N stretching), and 2959 cm^−1^ (C–H stretch). These positions are in good agreement with the typical secondary structure of Z. Several characteristic peaks belonging to the phenolic structure of Oxy are visible in its FTIR spectrum. A broad absorption centered near 3201 cm^−1^ corresponds to phenolic O–H stretching. The width of this band points to strong intra- and intermolecular hydrogen bonding, a typical feature of polyphenols that possess multiple hydroxyl groups. A sharp signal appears at 1669 cm^−1^, which is assignable to aromatic C=C stretching modes. This peak confirms that the benzene ring framework remains intact. At 1279 and 1173 cm^−1^, two moderately intense and well-separated bands are seen, corresponding to C–O stretches from phenolic–OH groups and possibly ester functionalities. Absorptions in the 1173–1040 cm^−1^ region are assigned to C–O–C and C–OH stretching. Bands in the fingerprint region (824, 737, 559 cm^−1^) correspond to C–H bending that occurs outside the molecular plane and skeletal vibrations of the aromatic rings. Alg exhibits a broad O–H stretching band around 3436 cm^−1^, a C–H stretching peak at 2923 cm^−1^, and strong absorptions at 1126 and 1097 cm^−1^ from C–O–C vibrations, which are characteristic of a carbohydrate backbone.

The formation of the Oxy-Z/Alg composites leads to distinct spectral variations across all mentioned regions. Compared with the individual components, the O–H/N–H stretching band (located at 3369 cm^−1^) widens and undergoes a slight red shift. This spectral behavior points to hydrogen bonding interactions involving Z, Oxy, and the hydroxyl groups of the Alg [36]. Amide I band exhibits a slight red shift and reduced peak height at 1649 cm^−1^. Meanwhile, Amide II band near 1540 cm^−1^ moves modestly downward in frequency. The C–O–C vibrations of the Alg broaden but keep their original intensity, pointing to effective incorporation into the composite materials while avoiding chemical degradation. The aromatic C=C and C–O peaks of Oxy become slightly weaker and shift by small amounts, reflecting non-covalent forces, potentially including π–π stacking and hydrogen bonding to the Z backbone [37]. These observations seem to indicate that the Oxy-Z/Alg composites maintain the structural integrity of the protein, polyphenol, and polysaccharide components while achieving stable integration, a feature that would be consistent with a rationally engineered carrier system.

#### 3.2.4. Secondary Structural Analysis

To further quantify the conformational changes in Z, the Amide I region (1600–1700 cm^−1^) was analyzed. It is important to recognize that the O-H bending of H_2_O can affect the absorbance near 1640 cm^−1^, where protein signals also appear, leading to possible overlap. Second-derivative analysis was used here to determine the exact sub-peak locations, thereby separating the Z’s secondary structure signals from the broad interference caused by remaining water. Gaussian curve-fitting was then used to estimate the fractional distribution of α-helix, β-sheet, β-turn, and random coil structures (Figure 5C,D).

The dominant structural motif in pure Z was the β-sheet, which contributed 37.20% to the entire conformational content (Figure 5B). This pattern is typical for Z when present in an aggregated or solid condition. Upon formation of the ternary complexes, a substantial reallocation of these structural components took place. A notable decrease in β-sheet content to 22.31% was observed, accompanied by an increase in α-helix from 22.84% to 27.96%. Meanwhile, β-turn content rose substantially, going from 17.79% to 29.91%. This observation may suggest that adding Oxy along with the polysaccharide could alter the self-association behavior of Z. A reduction in the rigid β-sheet fraction suggests that the original protein assemblies have been broken apart, resulting in a more flexible and restructured protein network. A higher proportion of α-helix segments points to ligand-induced stabilization and ordering of the conformation, presumably resulting from the creation of additional intra-chain hydrogen bonds. This change from a highly aggregated architecture to one that is more reorganized and stabilized could be essential for encapsulating the bioactive compounds, as it may reflect the adaptation of the Z’s secondary structure to accommodate the active molecule within the delivery system [38].

#### 3.2.5. DSC Analysis

**Figure 6:**
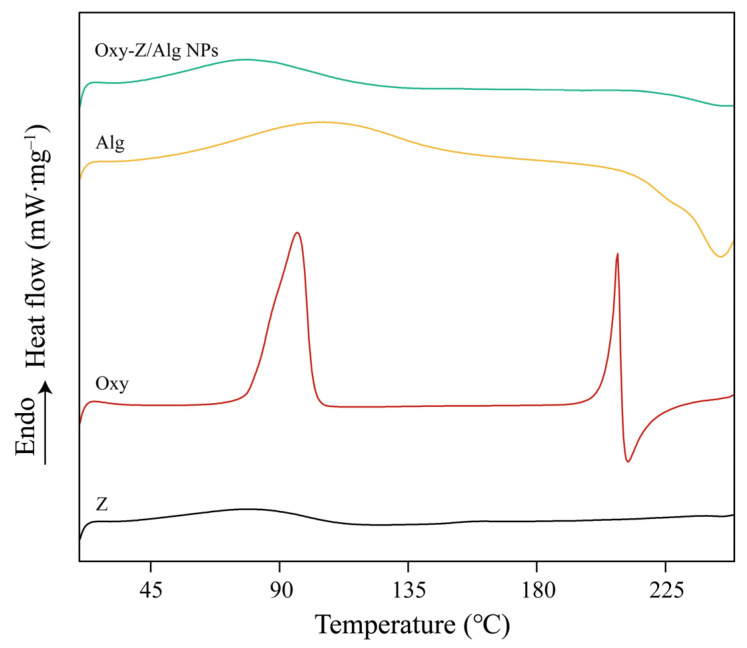
DSC analysis of Z, Oxy, Alg, and Oxy-Z/Alg NPs (prepared with a Z concentration of 5.0 mg·mL^−1^, an Oxy/Z mass ratio of 1:3.2, and a Z/Alg mass ratio of 5:1).

The thermal performance and crystalline state of Oxy located in the Oxy-Z/Alg complexes were characterized by DSC, as shown in Figure 6. In the thermogram of pure Oxy, a broad endothermic peak around 95 °C corresponds to dehydration of its crystal water content. And a distinct upward sharp feature at 209 °C indicates the endothermic fusion of the polyphenol crystals. Pure Z displayed a characteristic endothermic peak spanning 70 to 108 °C, which may be attributable to the denaturation of its structure caused by heating. A broad endothermic peak located around 102–148 °C was observed in the DSC thermogram of the Alg, which came from evaporation of water bound to the material. A subsequent decrease above 232 °C marked the start of thermal decomposition. Notably, the sharp endothermic melting peak of pure Oxy at 209 °C was not observed in the DSC curve of the Oxy-Z/Alg NPs. These results show excellent consistency with the findings obtained from XRD characterization analysis, suggesting a significant change in the physical state of Oxy or its high degree of dispersion within the biopolymer matrix. Additionally, compared with the individual materials, the broad endothermic band of Oxy-Z/Alg NPs located in the 70 to 108 °C region exhibited a slight shift toward higher temperatures, indicating a modified thermal resistance of the composite system. This state of molecular immobilization may indicate that the guest molecule is stabilized within the framework via non-covalent forces such as hydrophobic forces and hydrogen bonding, which effectively prevent the long-range ordering required for recrystallization [39]. To further study the underlying interaction mechanisms, molecular docking and molecular dynamics simulations were used to study the binding of Oxy to the Z/Alg NPs.

### 3.3. Molecular Docking Analysis

**Figure 7:**
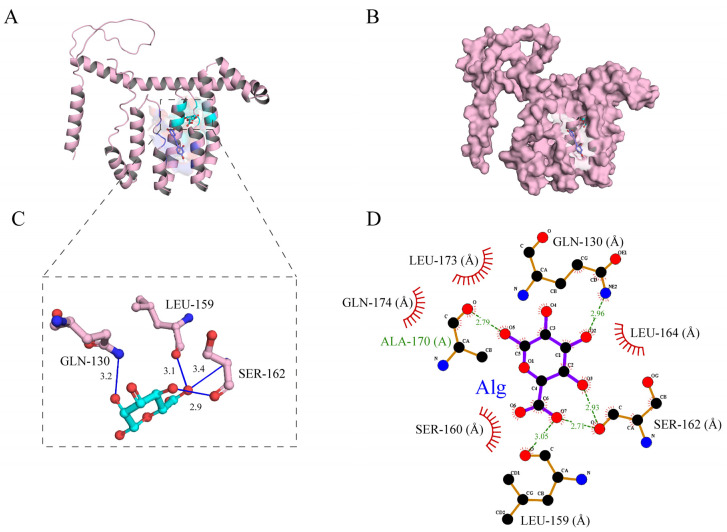
Molecular docking analysis of the Oxy-Z/Alg NPs. (**A**,**B**) 3D conformational representations showing the spatial orientation of ligands within the hydrophobic pockets of Z; (**C**) Magnified 3D view of the key residues at the binding interface; (**D**) 2D schematic of the intermolecular forces. Green dashed lines suggest hydrogen bonding, while red spoked arcs represent hydrophobic effects.

To study the interactions with the Oxy-Z/Alg ternary complexes, molecular docking was used for the first time to identify their binding characteristics. As shown in Figure 7A,B, the three-dimensional (3D) analysis revealed that Oxy was deeply embedded within the hydrophobic pocket of Z, stabilized by non-covalent interactions. Figure 7C shows that LEU-159, GLN-130, and SER-162 are key residues at the binding interface. These residues formed close contacts with Oxy at distances of 3.1, 3.2, and 3.4 Å, respectively, helping to stabilize its orientation. The two-dimensional (2D) interaction diagram (Figure 7D) shows the non-covalent interactions with the NPs. A hydrogen bonding network was observed, involving the carbonyl oxygen of ALA-170 (2.79 Å) and the side-chain nitrogen of GLN-130 (2.96 Å), contributing to the stability of the complexes. SER-162 and SER-160 also participated in hydrogen bonding, further stabilizing the complexes. Figure 7C shows that SER-162 formed two hydrogen bonds with A (with distances of 3.1 Å and 3.4 Å), helping to stabilize its orientation. The nearby SER-160 also contributed to the hydrophilic interface. Hydrophobic contacts from LEU-173, GLN-174, and LEU-164 (indicated by red spoked arcs in Figure 7D) formed a hydrophobic environment around Oxy. The high EE (78.35%) is likely due to van der Waals and hydrogen bonds involving serine and glutamine. Molecular docking showed that Alg bound to Z with an affinity of −5.03 kcal∙mol-1, suggesting that Alg binding induces a conformational change in Z’s pocket to accommodate Oxy.

### 3.4. Molecular Dynamics Simulations Analysis

We used molecular dynamics simulations to assess the stability of the Oxy-Z/Alg NPs via RMSD, RMSF, RG, SASA, and hydrogen bond analysis, following up on the docking results.

#### 3.4.1. RMSD Analysis

**Figure 8:**
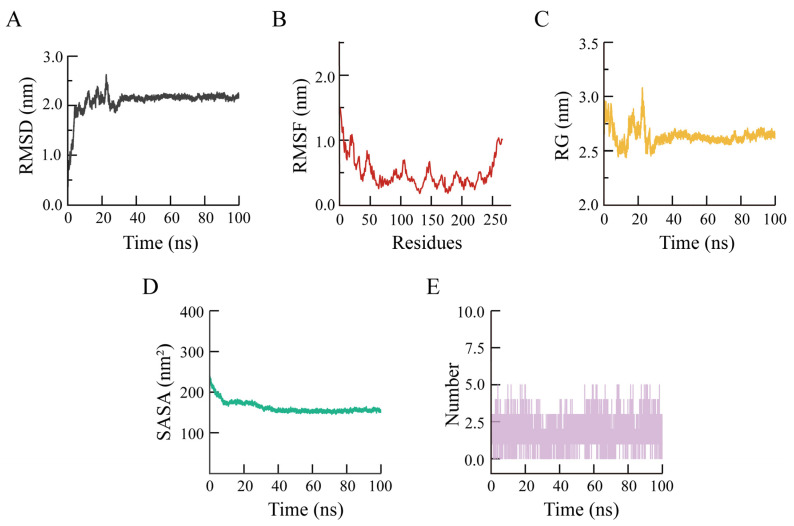
Molecular dynamics simulation results of Oxy-Z/Alg NPs. (**A**) RMSD analysis; (**B**) RMSF analysis; (**C**) RG analysis; (**D**) SASA analysis; (**E**) hydrogen bonding interactions.

The structural stability of the Oxy-Z/Alg complexes was first assessed using RMSD analysis. Figure 8A shows that the RMSD increased initially and then stabilized after 40 ns, fluctuating around 2.0 nm. This stable RMSD indicates that the complex reached equilibrium, with Alg and Oxy stably bound within Z, preventing further large conformational changes.

#### 3.4.2. RMSF Analysis

RMSF was calculated to assess the effect of ligand binding on Z flexibility. Figure 8B shows that the RMSF decreased after ternary complexes formation, indicating reduced backbone mobility. The RMSF values of binding pocket residues (SER-162, ALA-170, and GLN-130) were lower than those of other regions. This reduced flexibility suggests that these residues serve as key anchoring points, where non-covalent interactions limit fluctuations in Z.

#### 3.4.3. RG Analysis

As shown in Figure 8C, RG decreased during the first 25 ns, from 2.8 nm to a plateau at ~2.6 nm. This trend indicates that the complex became more compact over time, possibly due to hydrophobic interactions. The RG decrease shows that Z became more compact, likely enclosing Oxy in a hydrophobic environment.

#### 3.4.4. SASA Analysis

The SASA profiles in Figure 8D show a reduction in the solvent-exposed surface area, from 230 nm^2^ to 152 nm^2^. The lower SASA suggests that hydrophobic regions became buried, with polysaccharide chains protecting hydrophobic regions from water. The hydrophobic core protects Oxy from the aqueous environment, enhancing its stability.

#### 3.4.5. Hydrogen Bond Number Analysis

Hydrogen bonding frequency indicates of the stability of the complex and shows how these interactions maintain its structure. Figure 8E shows that the complexes formed 2–5 interfacial hydrogen bonds during the stable period, indicating steady affinity between the components. These hydrogen bonds, involving SER-162 (2.9–3.4 Å) and ALA-170 (2.79 Å), act like glue to hold the complex together. Hydrogen bonds and hydrophobic interactions together stabilize the delivery system and protect Oxy.

#### 3.4.6. Binding Free Energy Analysis

**Figure 9:**
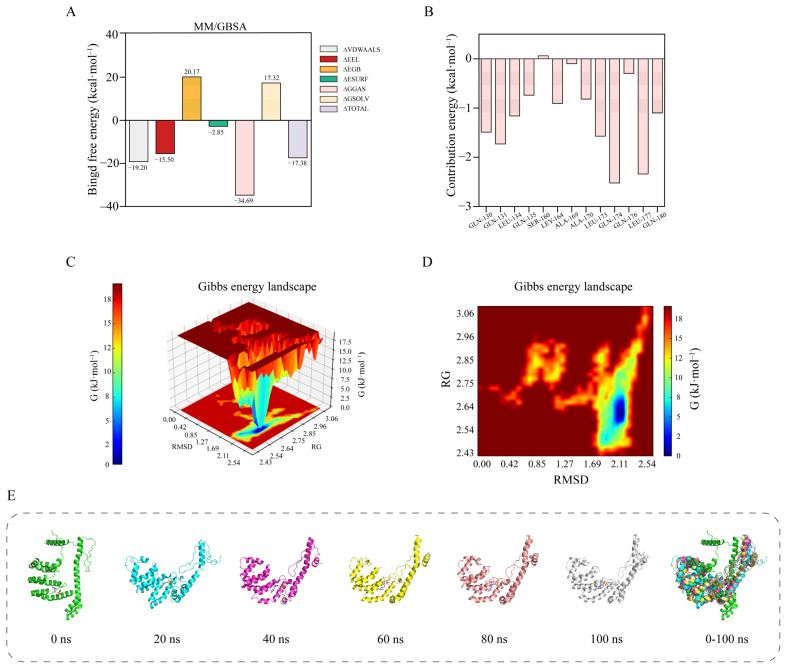
Binding affinity and stability of Oxy-Z/Alg NPs. (**A**) MM/GBSA energy. (**B**) Free energy evolution. (**C**,**D**) 3D and 2D free energy landscapes (FEL). (**E**) Time-resolved snapshots (0–100 ns) and superimposed structures, showing structural changes.

MM/GBSA method was used to determine the binding free energy (∆TOTAL) and its components for the Oxy-Z/Alg NPs. Figure 9A shows a negative binding free energy of −17.38 kcal·mol^−1^, indicating spontaneous binding and complex stability. Energy decomposition shows that van der Waals interactions (∆VDWAALS) contributed the most to the complex stability (−19.20 kcal·mol^−1^). This supports the hydrophobic collapse observed from RG and SASA, indicating that Z’s hydrophobic regions bind Oxy. Electrostatic interactions (∆EEL) also contributed −15.50 kcal·mol^−1^, due to hydrogen bonds between Alg hydroxyl groups and Z residues SER-162 and GLN-130. The gas-phase interactions (∆GGAS = −34.69 kcal·mol^−1^) overcame the desolvation cost (∆EGB = 20.17 kcal·mol^−1^), resulting in net favorable binding. We conclude that van der Waals and electrostatic forces promote spontaneous assembly, locking Oxy in the Z/Alg matrix and ensuring complex stability.

#### 3.4.7. Residue Energy Decomposition

To understand how specific residues contribute to NPs’ stability, we performed per-residue energy decomposition. Figure 9B identifies key residues responsible for the high affinity of the Oxy-Z/Alg complexes. GLN-174 and LEU-177 were the primary energy contributors, with free energy values of −2.52 kcal·mol^−1^ and −2.34 kcal·mol^−1^, respectively. GLN-174’s contribution is due to its role in forming hydrogen bonds and stabilizing the polar environment. The large contribution from LEU-177 and LEU-173 (−1.57 kcal·mol^−1^) highlights the role of hydrophobic burial in Z. Residues GLN-130 (−1.49 kcal·mol^−1^) and ALA-170 (−0.82 kcal·mol^−1^) also helped stabilized the complexes. These values are consistent with the docking distances from molecular docking, such as the 2.79 Å bond at ALA-170. The structural positioning of SER-162 within the binding interface (Figure 7D) suggests it contributes to the structural stability of the particles. And the 2.93 Å hydrogen bond and restricted mobility of this residue suggest its role in anchoring Oxy. The energy profile shows that localized polar interactions, embedded in a hydrophobic environment, stabilize the system rather than simple non-specific clustering.

#### 3.4.8. Free Energy Landscape (FEL) Analysis

To study the structural stability of the Oxy-Z/Alg NPs, 3D and 2D FEL were mapped using the RMSD and RG as coordinates (Figure 9C,D). The FEL provides the energy topography of the system’s conformational space. The landscape exhibits a funnel shape. The complexes pass through several metastable states (∆G > 12.5 kcal·mol^−1^) before the global minimum (∆G ≈ 0.0 kcal·mol^−1^). An energy basin at RMSD 2.11 nm and RG 2.14 nm is the global minimum, representing the most stable state of the NPs. The FEL results support the MM/GBSA finding that the complexes are stabilized by specific key residues. The deep energy minimum in the FEL confirms the high thermodynamic stability of the Oxy-Z/Alg complexes. The presence of a unique energy minimum confirms that the complexes form a thermodynamically stable and structurally consistent configuration under the simulated conditions.

#### 3.4.9. Conformational Dynamics Analysis

The 100 ns MD trajectory snapshots reveal the structural evolution of the Oxy-Z/Alg complexes over time. Figure 9E shows that within the first 20 ns, the Z backbone expanded slightly, allowing the binding of Oxy and Alg. The complexes became more compact between 40 and 60 ns, reaching their stable conformation. Snapshots from 80 to 100 ns show consistent structures, indicating that the complexes had reached the global minimum of the free energy landscape. The 0–100 ns superposition shows stable structures, especially in the central hydrophobic helices. The 0–100 ns superposition shows strong conformational overlap, especially in the central hydrophobic helices. The snapshots are consistent with the RMSD data, showing that the system both reaches equilibrium after 40 ns and forms a compact structure via hydrophobic collapse. The decrease in SASA (from 230 to 150 nm^2^) and RG (2.8 to 2.6 nm) indicates that hydrophobic domains become buried, which may protect Oxy from oxidation. The predicted intermolecular distances agree with the FTIR data, suggesting that the hydrogen bond reorganization shifts the amide and hydroxyl stretching frequencies within the ternary NPs. Conformational compaction prevents regular chain alignment, explaining the loss of crystallinity in the XRD patterns of the Oxy-Z/Alg complexes. Stable encapsulation restricts Oxy mobility, which matches the reduced desolvation heat from DSC. Our combined computational and experimental data reveal that hydrogen bonds and hydrophobic interactions drive the stability of the Oxy-Z/Alg system.

### 3.5. Stability of NPs

#### 3.5.1. Thermal Stability

**Figure 10:**
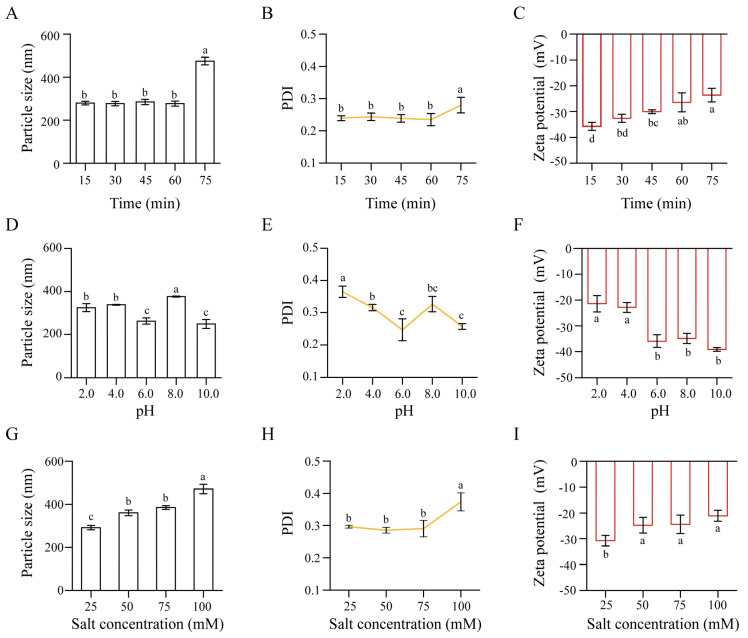
Stability of Oxy-Z/Alg NPs (prepared with a Z concentration of 5.0 mg·mL^−1^, an Oxy/Z mass ratio of 1:3.2, and a Z/Alg mass ratio of 5:1) under different conditions. (**A**–**C**) Change in particle size, PDI, and zeta potential after thermal stress (80 °C, up to 75 min). (**D**–**F**) Impact of particle size, PDI, and zeta potential across a wide pH 2.0–10.0. (**G**–**I**) Impact of particle diameter, PDI, and electrostatic charge at 0–100 mM NaCl. Data are presented as mean ± standard (*n* = 3). Different letters indicate significant differences (*p* < 0.05).

Thermal stability is important for colloidal delivery systems, as they may be exposed to elevated temperatures during processing. The effect of thermal stress (15–75 min) on the system was monitored by changes in particle size, PDI, and zeta potential. As shown in Figure 10A–C, the particle size fluctuated within 277–285 nm during the first 60 min of heating, and the PDI values remained below 0.24. These results indicate a uniform dispersion resistant to heat-induced aggregation. After 75 min of heating, the NPs showed a particle size of 475 nm. This expansion was accompanied by a zeta potential shift from −36 mV (15 min) to −24 mV (75 min). Despite some change in zeta potential, the PDI remained below 0.30 with no aggregation, suggesting the Alg layer stabilized the system. The Oxy-Z/Alg NPs maintained sub-micron size and colloidal stability after heating at 80 °C for 75 min, showing thermal resistance.

#### 3.5.2. pH Stability

The stability of the Oxy-Z/Alg NPs over a pH range of 2.0–10.0 was also assessed. As shown in Figure 10D–F, the NP’s size and zeta potential were pH-dependent across the tested range. Under pH 2.0, the NPs displayed a diameter of 325 nm with a zeta potential value of −21 mV. This phenomenon is due to the partial protonation of the carboxyl groups on Alg at this pH, which reduces the negative zeta potential and weakens electrostatic repulsion. The weaker repulsion led to an increase in particle size. The NPs were stable at pH 6.0–10.0, with the zeta potential reaching −39 mV at pH 10.0. This shift is likely due to deprotonation of the carboxyl groups in Alg and the negative charge on Z, keeping the system well-dispersed. When the pH exceeds the isoelectric point of Z (~6.0), the protein transitions from positive to negative charge, increasing its solubility and promoting assembly with the deprotonated polysaccharide [40]. The enhanced electrostatic repulsion led to a compact size of 249 nm at pH 10.0. The PDI remained below 0.33 across the pH range, suggesting that monodispersity was maintained. The Oxy-Z/Alg system remained stable across the tested pH range.

#### 3.5.3. Ionic Strength Stability

The influence of ionic strength on the NPs was assessed at NaCl concentrations of 25–100 mM. As shown in Figure 10G–I, the NPs achieved a uniform size distribution (292 nm) with a PDI of 0.30 under a saline environment (25 mM NaCl), and their zeta potential was −31 mV. The system adapted to the increasing ionic strength, with the particle size transitioning to a stable 361 nm at 50 mM NaCl. Even at the maximum NaCl concentration of 100 mM, the complexes remained stable without any precipitation. The system was adapted by forming a larger equilibrium structure (471 nm) while maintaining favorable PDI (0.37) and zeta potential (−22 mV) for colloidal stability. The ternary NPs were stable up to 100 mM NaCl, suggesting they could be suitable for use in some food systems [41].

### 3.6. In Vitro Antioxidant Activity

**Figure 11:**
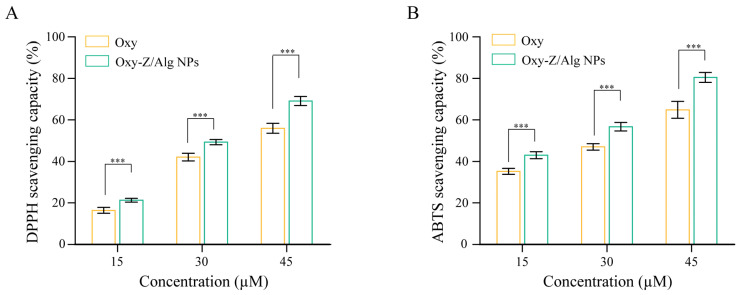
Radical scavenging of free Oxy and Oxy-Z/Alg NPs (prepared with a Z concentration of 5.0 mg·mL^−1^, an Oxy/Z mass ratio of 1:3.2, and a Z/Alg mass ratio of 5:1). (**A**) DPPH; (**B**) ABTS. Results are shown as mean ± SD (*n* = 3). Significant difference between free Oxy and Oxy-Z/Alg NPs at each concentration is marked by asterisks (*** *p* < 0.001).

The antioxidant performance of encapsulated Oxy was compared with that of free Oxy. As shown in Figure 11A, the DPPH scavenging activity of the Oxy-Z/Alg complexes increased from 21.31 ± 0.94% to 69.08 ± 2.20% as the concentration rose from 15 to 45 µM, outperforming free Oxy (16.44 ± 1.39% to 55.98 ± 2.34%). The ABTS assay showed a similar trend (Figure 11B). The NPs reached 80.43 ± 2.36% at the highest dosage, higher than the 64.84 ± 4.06% of free Oxy. The enhanced antioxidant activity is due to the good dispersion of Oxy in the Z/Alg matrix, which allows better interaction with radicals [42]. Unlike crystalline Oxy with low solubility, the Z/Alg complexes contain Oxy in an amorphous, molecularly dispersed state. The potent binding affinity (∆TOTAL = −17.38 kcal·mol^−1^) and site-specific anchoring at GLN-174 and SER-162 collectively restrict the conformational freedom of the Oxy, effectively trapping it in a disordered orientation throughout the molecular dynamics simulations. The Oxy-Z/Alg NPs maintain active hydroxyl groups at their surface, which improves Oxy’s dispersibility in water. This better accessibility may protect Oxy from degradation and ensures more Oxy is available for radical scavenging, consistent with the higher activity in the DPPH and ABTS assays.

## 4. Conclusions

In this work, natural biopolymers were employed to construct Oxy-Z/Alg NPs for the encapsulation and stabilization of Oxy, a potent antioxidant with poor aqueous solubility. Effective encapsulation relied on the hydrophobic regions of Z, and surface stabilization was achieved through the hydrophilic and polyanionic nature of Alg. XRD and DSC analysis indicated that Oxy was in an amorphous state within the NPs, while FTIR showed vibrational shifts, indicating strong intermolecular interactions. Molecular docking combined with molecular dynamics simulations showed that Oxy bound to GLN-174 and SER-162 via hydrogen bonds. This reconfiguration leads to hydrophobic collapse, with RG and SASA decreasing simultaneously, forming uniform nanostructures of ~243 nm. The integration of the anionic polysaccharide shell was indicated by a negative zeta potential (−36 mV). The system remained stable during heating (80 °C up to 75 min), over a pH range of 2.0–10.0, and at ionic strengths up to 100 mM NaCl, indicating their potential use in food or pharmaceutical formulations. The molecular dispersion of Oxy in the biopolymeric matrix increased its antioxidant activity with DPPH and ABTS inhibition rates of 69.09% and 80.43%, respectively. This work offers insights into the stability and self-assembly mechanism of the Oxy-Z/Alg complexes. These findings provide a theoretical basis for delivering Oxy with Z-based nanocomposites, and the resultant Oxy-Z/Alg NPs show promise for advancing therapeutic or functional food applications. This nanoformulation method also improved physicochemical properties, showing great promise for the further advancement of dietary supplements containing lipophilic ingredients.

## Data Availability

The raw data supporting the conclusions of this article will be made available by the authors on request.
